# Multiple recommended health behaviors among medical students in Western Canada: a descriptive study of self-reported knowledge, adherence, barriers, and time use

**DOI:** 10.3389/fmed.2024.1468990

**Published:** 2024-11-01

**Authors:** Nathanael Ip, Kendra Scarrott, Annalijn I. Conklin

**Affiliations:** ^1^Faculty of Pharmaceutical Sciences, University of British Columbia, Vancouver, BC, Canada; ^2^Faculty of Medicine, University of British Columbia, Vancouver, BC, Canada; ^3^Centre for Advancing Health Outcomes, Providence Health Care Research Institute, Vancouver, BC, Canada; ^4^Edwin S.H. Leong Centre for Healthy Aging, Faculty of Medicine, University of British Columbia, Vancouver, BC, Canada

**Keywords:** health behaviors, healthcare professionals, medical students, knowledge, adherence, barriers, time, prevalence

## Abstract

**Background:**

General medical practitioners are responsible for promoting and prescribing lifestyle modification and serve as role models for healthy behaviors. We aimed to assess self-reported knowledge, adherence, barriers and time spent on all recommended health behaviors among medical students.

**Methods:**

A cross-sectional online survey of eight behavioral domains among undergraduate medical students in The University of British Columbia, Canada, was analysed using descriptive statistics and visual display.

**Results:**

Between March and April 2023, 137 medical students participated in the survey (74% female). Over 80% had knowledge of five health behavior recommendations, but lacked knowledge of specific dietary recommendations in particular. Over 60% reported meeting guideline-recommended levels for tobacco, weekly alcohol, daily alcohol (females only), and physical activity (males only). Large gaps existed between knowledge and adherence for physical activity, sleep, sedentariness, screen time, and dietary recommendations. Sex differences in knowledge and adherence to recommended health behaviors were identified. Time spent on wellness focused on sleep (47–49 h/week), diet (9.6 h/week), exercise (5.8 h/week), and hobbies (6.1 h/week). Forgetting recommendations (69% of females, 71% of males), and lack of time (72% of females, 52% of males) were principal barriers to knowledge and adherence.

**Conclusion:**

Most medical students in Western Canada reported not meeting multiple recommended health behaviors. Time was the largest barrier to adherence and free time was spent on sleep. Medical education may require protected time and dedicated content for health behaviors to ensure future physicians can be role models of health promotion for patients.

## Introduction

It is widely accepted that adherence to health recommendations is essential for the prevention and management of diseases. Medical professionals serve as role models for healthful behaviors to their patients, with most patients expecting to be prescribed health behavior recommendations (“lifestyle modification”) by their physicians (1–5). Thus, students of medicine are expected to both know and adhere to key health recommendations through the achievement of physician competencies set by the Royal College of Physicians and Surgeons: namely, professional competency 4 (demonstrate a commitment to physician health and wellbeing to foster optimal patient care), leader competency 2 (engage in the stewardship of health care resources) and medical expert competency 1 (apply knowledge from the social/behavioral sciences) and 2 (select appropriate cost-effective interventions for health promotion in patient care) (6).

Current medical curriculum content on recommended behaviors for health promotion and disease prevention is, however, limited and patchy. There is inadequate coverage around Public Health and Preventive Medicine as important areas of knowledge for physicians, and the medical curriculum does not emphasize the scope, importance, or sources of public health recommendations in Canada. Instead, the syllabus introduces weekly disease themes wherein specific lifestyle modifications are mentioned piecemeal. For example, dietary recommendations for sodium intake are introduced during weeks focused on hypertension and heart failure, yet sodium is also relevant for kidney care and overall health. Similarly, recommended daily water intake is mentioned during a week focused on acute kidney injury but drinking adequate water is vital to many bodily functions including getting rid of wastes, protecting joints and organs and maintaining body temperature. Moderate intensity exercise is highlighted during weeks focused on hypertension, type II diabetes, and cardiovascular disease, although exercise is also important for sleep, mood, cancer, bone health and obesity. Since the medical education program structure teaches selected health behavior recommendations exclusively through a patient pathology lens, it lacks a holistic health maintenance framework to educate medical students on the comprehensive set of health behavior recommendations from Health Canada. In addition, the medical program structure provides little to no personal time for extracurricular activities of medical students including self-care and health-promoting behaviors; this deficit is highlighted by the rising prevalence of burnout among Canadian physicians and medical residents from a third in 2018 to more than half in 2021 (7, 8). It is a public health and occupational imperative that medical students learn about and develop protective and sustainable health behaviors for their future health, the sustainability of the healthcare system and most importantly for their role modelling for patients.

Globally, a body of literature suggests that a significant proportion of medical students fail to meet important health behavior recommendations. For example, one study showed that about a quarter of all medical students surveyed reporting sleeping less than recommended levels (9), and multiple studies reported that a majority of medical students did not achieve the evidence-based recommendations for sleep (10, 11). Several studies have also shown the high prevalence of tobacco use and alcohol consumption among medical students, contrary to health guidelines (12–14). Medical students in the US, however, appear to meet or exceed recommendations for physical activity (15–19). One study in Canada also showed that medical students engaged in more strenuous physical activity when compared to the general population (20). Despite some research on this topic, no research has comprehensively assessed knowledge, barriers, adherence and time-burden of all health-promoting behaviors, including dietary recommendations, among medical students. Prevalence studies on specific behaviors also rarely consider the reasons medical students fail to meet health guidelines even when students possess clear knowledge of recommended health behaviors.

In a departure from literature, this study aimed to fill a knowledge gap by comprehensively assessing self-reported knowledge, adherence, barriers, and time spent on all recommended health behaviors among medical students in Western Canada. This research expands current evidence on the prevalence of all health behaviors among physicians-in-training and adds new data on the estimated time burden and barriers of adherence.

## Materials and methods

### Subjects and data collection

A cross-sectional survey was administered between March and April 2023 to University of British Columbia (UBC) medical students in all 4 years of the undergraduate Medical Program from the classes of 2023–2026 across all Medical Program sites of UBC (*n* = 1,152): the medical student population is 59% female and 80% aged 21–26 years. The online semi-structured survey collected anonymous data from 137 respondents through the institutionally approved Qualtrics platform, and included predetermined and free-text response categories. The questionnaire administered 30 items across demographics, self-reported knowledge of recommendations for personal application, achievement of recommendations, and hours per week spent on 8 behavioral domains (Supplementary Methods). Health behavior guidelines often vary by individual, depending on factors such as age (as with sleep) and body mass (as with water) (21, 22). For this reason, students’ knowledge of their health behavior recommendations could not be directly tested. Instead, students were asked to self-report whether they felt they knew how each recommendation applied to them. The questionnaire was developed based on clinical guidelines and government websites with health behavior recommendations (e.g., https://www.healthlinkbc.ca/), and was piloted with 2 medical students and 1 health professional known to the research team. This research was approved for minimal risk by the UBC Behavioral Research Ethics Board (H16-00044), and written informed consent was obtained from survey participants.

### Data cleaning and analysis

Responses were excluded if participants answered fewer than four survey questions beyond the demographics section (*n* = 20), resulting in a final sample size of 117 for analysis (about 10% of the medical student population in the province; Supplementary Figure S1). Open-text data were coded for quantitative content analysis and frequencies (proportions) were used to analyze knowledge and adherence. Surveys were analyzed in Microsoft Excel v16.72. Responses to the hours per week section were analyzed using MATLAB vR2022b. The frequency of the knowledge and achievement of barriers was summarised in bar graphs by sex; results for average hours per week spent on a recommended health behavior were reported by medical year.

## Results

The majority of survey respondents were medical students who self-identified as female (74%), with an even distribution across medical year 1 (19%), year 2 (30%), year 3 (32%) and year 4 (20%). Most survey respondents were from the Vancouver Fraser Site (39%) or the Southern Site (38%), with respondents from the Interior Site (9%) and Northern Site (15%) accounting for around a fifth of all respondents (Supplementary Table S1).

**Figure 1:**
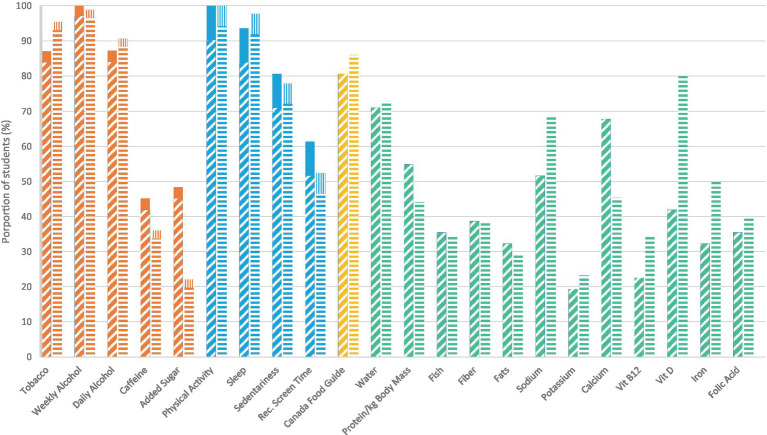
Prevalence of self-reported behaviors of health behavior recommendations among medical students in Western Canada, by sex. Hash, male respondent; solid, male no response/skipped; horizontal stripe, female; vertical stripe, female no response/skipped; *n* = 117.

The prevalence of self-reported knowledge of health behavior recommendations varied widely by behavior, with similar proportions of male and female medical students reporting having knowledge of nearly two-thirds of the measured behaviors (Figure 1). More males than females skipped questions about knowledge of physical activity, sleep, sedentariness and screen time. Most respondents reported having knowledge of recommendations for weekly alcohol (96% of females, 97% of males), physical activity (94% of females, 90% of males), daily alcohol (88% of females, 84% of males), tobacco (93% of females, 84% of males), sleep (92% of females, 84% of males), and Canada’s Food Guide (86% of females, 81% of males). Some notable sex/gender differences were seen for specific dietary recommendations. A majority of female medical students reported knowing the recommendation for Vitamin D (80%), but less than half of male medical students reported knowing the recommendation (42%). Most of the female and male medical students reported having knowledge of recommended daily water intake (72% of females, 71% of males), more female (69%) than male (52%) medical students reported knowing about sodium recommendations. Less than half of respondents reported knowing the recommendations for caffeine and added sugar, again with notable sex/gender differences: 45% of males reported knowing the recommendation for added sugar (45%) but only 20% of female respondents reported knowing the recommendation. Knowledge was also reported to be less prevalent among medical students for dietary guidelines on calcium (45% of females, 68% of males), fiber (38% of females, 39% of males), fats (29% of females, 32% of males), and potassium (23% of females, 19% of males).

**Figure 2:**
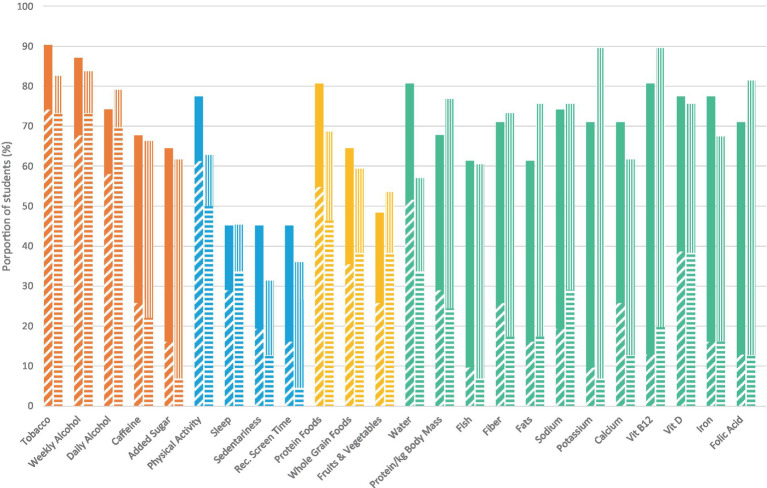
Prevalence of meeting recommended health behaviors among medical students in Western Canada, by sex. Hash, male; solid, male no response/skipped; horizontal stripe, female; vertical stripe, female no response/skipped.

As shown in Figure 2, over 60% of male medical students met the health recommendations for tobacco, weekly alcohol intake and physical activity and over 60% of female medical students met the recommendations for tobacco, weekly and daily alcohol intake; less than half of female medical students met the recommended physical activity levels. About half of male medical students (52%) met the recommendation for water, whereas a third of female medical students achieved this health behavior (34%). Adherence of respondents to the other non-dietary behaviors was generally low across male and female respondents, with some differences by sex/gender in meeting the recommendations for sleep (34% of females, 29% of males), sedentariness (13% of females, 19% of males), recreational screen time (5% of females, 16% of males). In free-text responses, medical students reported that they did a behavior when it gave them a noticeable positive effect: females most often listed exercise and sleep and males most often listed as exercise and hobbies (e.g., “I take time for hobbies because I think it prevents burnout”).

There was wide variation in the proportion of medical students meeting dietary recommendations. Female and male medical students were similar in meeting the recommendations for whole grain foods (38% of females, 35% of males), fish (7% of females, 10% of males), fats (17% of females, 16% of males), potassium (7% of females, 10% of males), vitamin D (38% of females, 39% of males), iron (16% of females, 16% of males), and folic acid (13% of females, 13% of males). However, other dietary recommendations were not met equally: fruits and vegetables (38% of females, 26% of males), protein (47% of females, 55% of males), fiber (17% of females, 26% of males), calcium (13% of females, 26% of males), and sodium (29% of females, 19% of males).

**Figure 3:**
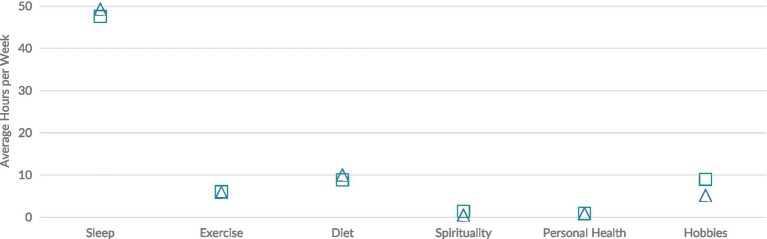
Reported time spent to achieve recommended health behaviors among medical students in Western Canada, by sex. Triangle, female; square, male.

As Figure 3 illustrates, respondents spent the most time on achieving sleep recommendations. Females reported spending an average of 49.29 h per week on sleeping, while males reported spending an average of 47.54 h per week on sleeping. Students reported spending an average of around 10 h a week adhering to dietary guidelines (10.03 h for females, 8.87 h for males) and around 6 h a week adhering to exercise guidelines (5.86 h for females, 6.07 h for males). Male respondents reported spending almost double the average amount of time (8.98 h) on hobbies when compared to female respondents (5.19 h). Students spent significantly less time on spirituality (approximately 1 h/week) and personal health (approximately 1 h/week).

**Figure 4:**
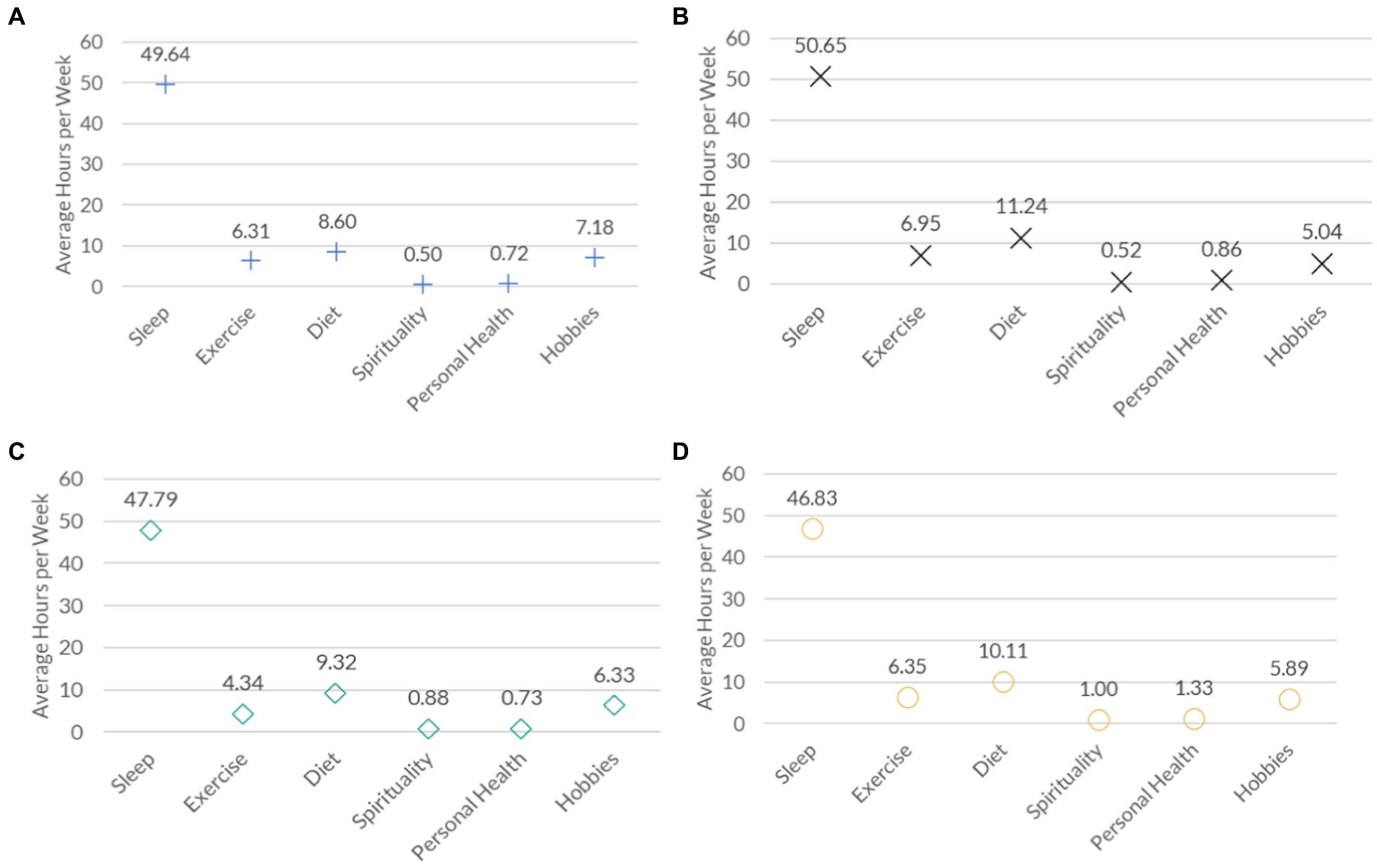
Reported time spent to achieve recommended health behaviors among medical students in Western Canada, by year. Panel A, year 1; Panel B, year 2; Panel C, year 3; Panel D, year 4.

Figure 4 shows some variations across medical years in the time spent on achieving recommended health behaviors. In particular, year 1 medical students (Panel A) spent more time on sleeping (49.64 h/week) and hobbies (7.18 h/week) than year 4 medical students (respectively, 46.83 h/week and 5.89 h/week) (Panel D). By contrast, year 1 medical students spent less time on diet (8.60 h/week), spirituality (0.50 h/week) and personal health (0.72 h/week) than did year 4 medical students (10.11 h/week; 1.00 h/week; 1.33 h/week). Differences in time spent on activities across medical years was not statistically significant (ANOVA *p* = 0.566).

About 10% of respondents reported that they had achieved the recommendations for diet but were unable to estimate the time, but less than 5% of medical students reported meeting recommendations without estimating the time spent on each behavior (Supplementary Figure S2). Between 20 and 30% of medical students did not respond to questions about time spent on health behaviors.

**Figure 5:**
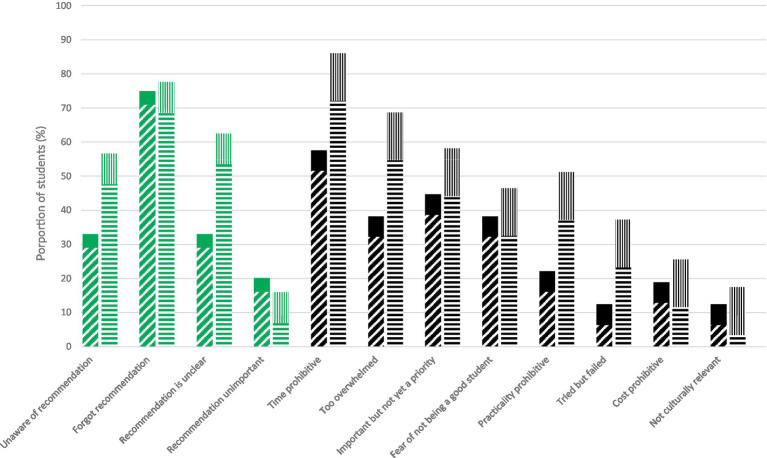
Prevalence of barriers to knowledge (green) and behaviors (black) among medical students in Western Canada, by sex. Hash, male; solid, male no response/skipped; horizontal stripe, female; vertical stripe, female no response/skipped.

The most common barrier to knowledge of recommended health behaviors was forgetting the recommendation in both female (69%) and male (71%) medical students (Figure 5). Around half of female medical students reported they were unaware of the recommendation (48%) or it was unclear (53%), whereas 29% of male medical students were unaware of the recommendation or indicated it was unclear. A small proportion of respondents indicated that the recommendation was not important (7% of females, 16% of males). Achieving recommended health behaviors was time-prohibitive for 72% of female medical students and 52% of male medical students. A higher proportion of female medical students reported a range of barriers to achieving recommended health behaviors than males. Key barriers to achieving health behaviors included: being too overwhelmed (55% of females, 32% of males), the behavior was important but not a priority (44% of females, 39% of males), and the behavior was not practical (37% of females, 16% of males). Both females and males shared other barriers to the recommended health behavior such as a fear of academic decline (33% of females, 32% of males), cost (12% of females, 13% of males) and lack of cultural relevancy (3% of females, 6% of males). In free-text responses asking about barriers to health behavior adherence, female respondents specifically identified two time-consuming themes of being a caregiver/parent and being responsible for housework that male respondents did not report as time constraints. Table 1 provides examples of free-text written responses from female and male medical students about the barriers to meeting recommended behaviors.

**Table 1:** Narrative responses to free-text questions about barriers to meeting recommended health behaviors.

Survey respondent group	Examples of written responses about barriers to meeting recommended health behaviors
Female medical students	“If I spend more time on diet and preparing meals this means less time for thinks like sleep and exercise” “I may have the time, but I am so exhausted when I am done the day in clerkship I just cannot bring myself to exercise, or I forget to eat.” “I want to exercise but I am so tired from clerkship and my mind is so dead that I just lay in bed.” “Too many conflicting health behaviours to consider at once—e.g., too many aspects of dietary intake to think about” “I would REALLY like to cut down my time cooking/cleaning.” “Between being a student and caregiver, I just do not have the time or support to focus on myself. It makes me feel guilty comparing myself to my classmates who seem better able to prioritize themselves.” “I spend a lot of time cooking and cleaning but still will eat takeout and frozen meals when I run out of meal prep.” “I feel negatively about the amount of time I need to spend cooking and preparing meals because this is an added stress and I am looking for ways to shorten this. i am chronically sleep deprived as I am also a parent in medical school and I lack the energy and time to exercise as much as I want to.” “I wish I had more time for hobbies, but I feel pressured to do school work or run necessary errands during my non-committed time.”
Male medical students	“The only behaviour I’m happy about is my time with hobbies. It’s much less than I’d like still but it the bare minimum I need to avoid depression, and sometimes it’s not enough.” “I am happy to spend so much time prioritizing my hobbies and ensuring I cook and eat healthy and enjoyable food.” “I am constantly frustrated with this program a lack of time I get to spend doing things I like.” “I often do not sleep enough at night because I feel that I need to stay up and study for school.” “I sometimes would rather be studying than taking care of myself.” “I feel guilty about taking time away from studying to do almost anything else.” “The main reason I do not participate more with dietary tasks and the other categories that I value is the fear of being behind with school work.”

## Discussion

This cross-sectional study aimed to survey the self-reported knowledge, adherence, and barriers of health behaviors among medical students studying in Canadian institutions. We found that a large proportion of medical students reported having knowledge of five health behaviors but reported lacking knowledge of recommended levels for most other health behaviors, especially specific dietary factors. The prevalence of meeting the recommendations varied across health behaviors and large gaps were seen between knowledge and adherence for physical activity, sleep, sedentariness, screen time, and healthy eating practices in line with Canada’s Food Guide. Differences between the proportion of male and female respondents that reported knowledge of, and adherence to certain health recommendations were identified. Apart from sleep, the most amount of time spent on health behaviors was reported for diet and physical activity in both males and females. Across medical years, time spent on some behaviors decreased while other increased. Forgetting recommendations was identified to be the most prevalent barrier to knowledge, while lack of time was the most prevalent barrier to achieving the recommended behavior.

### Findings in relation to other work

We found that a large majority of respondents reported knowledge of guidelines for alcohol, tobacco, physical activity, healthy eating and sleep for their personal use. Our finding of high prevalence of reported knowledge on sleep recommendations in both male and female medical students is similar to, and higher than, other survey data from the United States which showed that 72% of medical students had correct knowledge about the recommended amount of 7–9 h a day for adults from guidelines of both the Canadian Society for Exercise Physiology and the American National Sleep Foundation (9, 21, 23). It is not surprising that we found over 85% of medical students in Western Canada reported having knowledge of recommendations for both tobacco or alcohol which are substances that have strong national and provincial public health campaigns over many decades, and have known harms (24–28). Equally, knowledge of physical activity recommendations and the new Canada Food Guide was also reported to be prevalent in BC medical students which has also been reported elsewhere in limited literature on the dietary and physical activity knowledge of healthcare providers in Canada and internationally (29–31). However, we found a reported lack of knowledge of the guidelines for other stimulants such as added sugar and caffeine, and specific dietary factors such as fish, fiber, fats and key micronutrients. One study found that between 8 and 26% of Australian postgraduate medical students surveyed had knowledge of the guidelines for sodium, mirroring the low reported prevalence of knowledge about sodium guidelines recorded in this present study (19% of male respondents, 29% of female respondents) (32).

The majority of respondents reported meeting recommendations for tobacco and weekly alcohol, with sex differences in adhering to recommended daily alcohol (female only) or physical activity (males only). These results are comparable to a similar study that found the large majority of surveyed American medical students reported never having smoked a cigarette (15). However, despite the fact that almost all medical students in Western Canada reported knowledge of physical activity and sleep guidelines, only around a half reported meeting physical activity recommendations and a about a third reported they achieved the recommendations for sleep. It is possible that respondents had a high level of self-reported knowledge for both behaviors due to wider public health campaigns aiming to increase awareness of recommended physical activity (ParticipACTION) and sleep (the Canadian 24-Hour Movement Guidelines, 24HMG) (23). A Public Health Agency of Canada paper found that 82% of Canadians surveyed recognized the ParticipACTION campaign when prompted while another group of researchers found a third of Canadians surveyed were aware of the 24HMG campaign (33, 34). However, the practical implementation of this knowledge did not match the level of knowledge which is likely to do the multiple barriers that medical students identified. Another reason for this large gap between knowledge and adherence of respondents in this present investigation is that behavior change requires more than awareness and information; it needs motivation and skills to perform the behavior in the context of a system of structured support (35, 36). Notably, Canada lacks physical activity and sleep interventions that provide behavioral skills or target barriers to guideline adherence. Motivation alone is complex and involves inter-linked factors (risk perception, action self-efficacy and outcome expectancies) that lead to intention for and maintenance of a recommended behavior (37). Some of the responses suggested that the gap between knowledge and behavior may simply be a consequence of a lack of any observable positive result.

Overall, medical student reported a low level of meeting the recommendations for fruits and vegetables (FV) which is national statistics and existing literature. Several studies concluded that medical students consume less than the World Health Organization (WHO) recommendation of five daily servings (38–40). Our study found that a third of medical students met FV guidelines, which is higher than previous research showing about one-tenth of medical and pharmacy students in California met the recommendations for FV (41). Notably, we also observed a sex/gender difference in this dietary behavior with 39% of females but 26% of males consuming the recommended levels of FV, which was also reflected in the other California survey of 12% of female and 8% of male respondents meeting FV recommendations (41). It is known that there are sex/gender differences in dietary intake, particularly FV, which may be attributed to a difference in attitudes towards FV consumption between males and females (42–46). Although about 40% of medical students reported knowledge of recommended fiber levels, we found that it was less common for medical students to meet fiber recommendations with some sex/gender differences. Previous qualitative research shows adherence to fiber guidelines of medical students is generally low (41, 47). One study surveying the nutrient intake of medical students in Greece found the mean daily consumption of fiber to be 16.9 grams in males and 13.7 grams in females, falling below the WHO/Food and Agriculture Organization and European Food Safety Authority recommendations of 25 grams a day fiber intake for adults (47–49). These results corroborate our study showing a higher proportion of male medical students (26%) than female medical students (17%) met the Canadian guidelines for fiber. Similarly, although about a third of medical students reported having knowledge of fish guidelines (35% of females and males), less than 10% reported being able to achieve recommended fish intakes (8% of all students), particularly female medical students (7%). The low rates of adherence to fish intake guidelines is reflective of the trend in the fish consumption of Canadians, with one study finding that the average Canadian failed to meet the recommendations for daily fish intake (50). However, the findings of this present study were surprising as British Columbia’s geographical location bordering the Pacific Ocean supports a large seafood industry that accounted for $1.4 billion in exports in 2018 (51, 52).

Achievement of recommendations for healthy behaviors can be particularly challenging to medical students due to logistics alone. Some examples include daily physical activity recommendations, daily sedentary time, sleep and nutrition. In addition to 150 min per week of moderate to vigorous aerobic physical activities, Canada’s 24-h movement guidelines recommend “several hours per day of light physical activity, including standing” as well as “limiting sedentary time to 8 h or less” which includes “no more than 3 h of recreational screen time” and “breaking up long periods of sitting as often as possible” (53). Medical training can require 3 to 5 days per week spent in traditional classroom or small-group lessons for 4 to 8 h. This program structure is particularly concentrated in the earlier, less-clinical years of training, and presents both physical and social constraints to engaging in disease-preventing physical activity and reducing large amounts of sedentary time. Requirements in the later, more clinical years of training present their own logistical barriers to Canada’s 24-h movement guidelines (54, 55). For example, “getting 7 to 9 h of good-quality sleep on a regular basis, with consistent bed and wake-up times” is not compatible with compulsory rotations requiring student availability on hospital wards for 27.5-h periods. These requirements also increase barriers to recommended caffeine intake and healthy eating choices.

As this study aimed to understand the feasibility of medical students achieving all the recommendations for health behaviors, our survey showed that medical students spend an average of 72 to 73 h per week on all health behaviors in their extra-curricular time. The most time was spent on sleep which had a non-significant decline by 2.8 h/week between year 1 and year 4. About 10 h per week were spent on average on achieving dietary recommendations and around 6 h per week were spent on physical activity and on hobbies. Both male and female medical students reported similar amounts of time spent on each of the behaviors. One study surveying the leisure-time physical activity of Canadian medical students has found students spend at least 15 min on exercise an average of 16.2 times per week, that is, that students spend an average of at least 4.05 h per week on physical activity. In comparison, our findings showed a slightly higher average of 6 h per week on physical activity (56). This inconsistency can likely be attributed to a difference in the method used to measure time spent on physical activity as Babenko and colleagues recorded the average number of times a week that students spent at least 15 min on exercise while this study measured the average time in hours per week directly (56).

Finally, a number of different barriers to both knowledge and adherence of multiple recommended health behaviors were identified. Forgetting recommendations was reported as the most prevalent barrier to knowledge, while lack of time was the most prevalent barrier to achieving the behavior. As reported by Nelson and colleagues 47% of American medical students surveyed found time to be a barrier to meeting physical activity guidelines while 17% of respondents found cost to be barrier to meeting recommended food guidelines (57). These findings mirrored our results with time representing a major barrier to recommended behaviors and cost representing a less common barrier to meeting recommendations for both female (12%) and male (13%) medical students. The current medical school policy schedules students for 40 h of curricular time per week, similar to that of a regular work week. Of these 40 h, 4–8 h of unstructured time is included for students to use at their discretion. However, medical students are also expected to commit a significant amount of time outside the aforementioned 40 h to study curricular work. One group of researchers found that working more than 40 h was associated with a negative impact on healthy eating in young adults (58).

An important finding was the sex difference in the reported barriers to adherence of recommended behaviors. Nearly three quarters of female respondents reported time as a barrier whereas only about half of male respondents noted time as a barrier to achieving health behaviors. In sum, female written responses indicated that sleep and exercise were already prioritised and, despite knowing other recommended behaviors are important, their bandwidth was full because they spend much of their time cooking/cleaning/running errands/being a caretaker; that is, females would have to trade off sleep and exercise in order to implement any other health behaviors in the context of non-curricular housework. Globally, time poverty is a gendered phenomenon whereby women are more time poor than men due to the gendered division of domestic labour (59). Among young workers it is shown that young females are responsible for greater amounts of housework than are young males (60). The barrier of time constraint may also explain the observed sex difference in other barriers to adherence, with more females reporting being too overwhelmed and being impractical to implement behaviors. The narrative responses showed that female medical students faced time barriers to adherence due to being a caregiver/parent and being responsible for housework such as cooking, cleaning and shopping. Research shows that not only do women spend more of their time on unpaid labour, but also that unpaid labour is correlated with negative mental health effects (61). Thus, our study results corroborate the broader literature on the uneven distribution of housework and reveal that both time and being overwhelmed are unique barriers to achieving health behaviors for female medical students who must balance the competing demands of both their curricular work and disproportionate housework, with the recommended health behaviors. These findings have implications for embedding gender equity in the physician competencies of the medical program and may require gender-specific considerations and structural interventions for more protected time for female medical students to serve as future role models for their patients.

### Methodological considerations

Limitations of our study warrant discussion. Our survey is limited by sample size and possibly volunteer selection bias that may underestimate the knowledge and adherence of health behaviors among Canada’s future clinicians from the West Coast. Our study did not offer incentives nor protected time for completion which likely affected the response rate; also, recruitment for our a two-month survey included only one reminder and was done through the newsletter of the Medicine Undergraduate Learner Access Advisory Council. Notably, medical students in Canada are known to have the lowest response rates to surveys relative to medical residents and practising physicians (62, 63). Additionally, our survey respondents in [*redacted for review*] were mostly female and thus findings may not be generalizable to male physicians-in-training or to other settings in Canada. However, our sample largely reflects the overall medical student pool as about 60% of all undergraduate medical students in the classes of 2023–2026 self-reported as female. While our collection method was expected to select females and males with equal sampling probability, it is well-known that females have higher survey response rates than males. To rebalance the under-representation of males in survey responses, future work may need to employ techniques such as a dual-frame sampling design to oversample males as a minority population of medical students in Western Canada.

A major strength of this study is the comprehensive inclusion of all recommended health behaviors, including water, sleep and sedentary behavior. To the best of our knowledge, this is the first study investigating the self-reported knowledge of, adherence to, time spent and barriers to the full breadth of health behaviors among future doctors in Canada. Another strength of this study is the focus on medical students who are an under-studied population of healthcare providers. This focus is imperative not only as physician burnout rates are increasing, but also as physicians should strive to serve as positive health role models for their patients (7, 8). Emphasized in physician competencies outlined by the Royal College of Physicians and Surgeons, Canadian medical students are expected to know and adhere to health behaviors by engaging in the stewardship of health care resources (leader competency); demonstrating a commitment to physician health and wellbeing to foster optimal patient care (professional competency); and, applying knowledge of social/ behavioral sciences and selecting interventions for prevention and health promotion in patient care (medical expert competency) (6). Of significance, the study’s novelty is the new data generated on estimated time spent on recommended health behaviors by way of assessing feasibility of medical students achieving health and wellness for themselves and modeling for their patients. Finally, another major strength of this study was a survey sample that consisted of a relatively equal balance of respondents from all 4 years of medical school, allowing results to be more representative of the general medical student population in Canada.

## Conclusion

The knowledge of and adherence to health behaviors are requirements for medical students who are expected to serve as role models for their future patients. This study demonstrated that medical students in Western Canada reported having knowledge of some but not all recommended health behaviors and that most did not achieve health promotion guidelines. This research also illustrated the average time required to achieve recommended health behaviors as well as identified a range of barriers to both knowledge and adherence, with forgetfulness and time being key barriers of each. There is great scope to improve the knowledge and adherence of recommended health behaviors among medical students, and a need to address relevant barriers.

## Data Availability

The raw data supporting the conclusions of this article will be made available by the authors, without undue reservation.
