# Supplementary material for: Multiple recommended health behaviors among medical students in Western Canada: a descriptive study of self-reported knowledge, adherence, barriers, and time use
Source: Front Med (Lausanne). 2024 Nov 1;11:1468990. doi: 10.3389/fmed.2024.1468990 (PMC11568874; doi:10.3389/fmed.2024.1468990)
Supplement: Supplementary file 1 [file Data_Sheet_1.docx]

**Supplemental Material - Results**

**Supplementary Figure 1**. **Flow diagram of study sample selection**

**Supplemental Table S1. Basic demographics of study sample**

| **Characteristic, n=117** | **Frequency (%)** |
| --- | --- |
| Sex, Female | 86 (74%) |
| Medical year of study |  |
| Year 1 | 22 (19%) |
| *Year 2 | 35 (30%) |
| *Year 3 | 37 (32%) |
| *Year 4 | 23 (20%) |
| Geographic location |  |
| Interior Site | 10 (9%) |
| Northern Site | 17 (15%) |
| Southern Site | 44 (38%) |
| Vancouver Fraser Site | 46 (39%) |

*Current year or on leave after finishing previous year.

**Supplementary Figure 2**. **Respondents for reported time spent to achieve recommended health behaviours among medical students in Western Canada.** Hash, answered (responded with an estimate); solid, responded but unable to estimate number; vertical stripe, no response
