# Supplementary material for: Multiple recommended health behaviors among medical students in Western Canada: a descriptive study of self-reported knowledge, adherence, barriers, and time use
Source: Front Med (Lausanne). 2024 Nov 1;11:1468990. doi: 10.3389/fmed.2024.1468990 (PMC11568874; doi:10.3389/fmed.2024.1468990)
Supplement: Supplementary file 2 [file Table_1.DOCX]

**SUPPLEMENTARY MATERIAL - METHODS**

| Feasibility of Recommended Health Behaviours for an Undergraduate Medical Student in British Columbia | | | | | | | | | |
| --- | --- | --- | --- | --- | --- | --- | --- | --- | --- |
| **Participant Demographics** | | | | | | | | | |
| 1. What is your distributed site? | | | | | | | | | |
| IMP | | NMP | | | SMP | | | VFMP | |
| 1. What is your current year of study? | | | | | | | | | |
| 1 | 2 | | | 3 | | 4 | | | I’m on leave |
| 1. If you are currently on leave, what is the last year you completed? If you are not currently on leave, please respond with “Not Applicable”. | | | | | | | | | |
| 1 | | 2 | | | 3 | | | Not Applicable | |
| 1. How did you identify yourself on your UBC medical school application? | | | | | | | | | |
| Male | | | Female | | | | [free response] | | |

| **Part 1:** The following questions relate to your knowledge of recommendations for important health behaviours for British Columbians. Some recommendations depend on a person’s age, stage of life, sex/gender, and health status. You can respond with yes, no, or not applicable. | | | | | | |
| --- | --- | --- | --- | --- | --- | --- |
| The following questions relate to diet. | | | | | | |
| 1. Do you feel that you know the recommended daily water requirements as they apply to you? | | | | | | |
| Yes | | | No | | Not Applicable | |
| 1. Do you feel that you know the Canada Food Guide daily food (fruits and vegetables, protein foods, whole grain foods) recommendations as they apply to you? | | | | | | |
| Yes | | | No | | Not Applicable | |
| 1. Do you feel that you know the following recommended daily nutrient requirements as they apply to you? | | | | | | |
| 1. Calcium | Yes | | | No | | Not Applicable |
| 1. Fats | Yes | | | No | | Not Applicable |
| 1. Fiber | Yes | | | No | | Not Applicable |
| 1. Folic Acid | Yes | | | No | | Not Applicable |
| 1. Iron | Yes | | | No | | Not Applicable |
| 1. Potassium | Yes | | | No | | Not applicable |
| 1. Daily protein per kg of body weight | Yes | | | No | | Not Applicable |
| 1. Sodium | Yes | | | No | | Not Applicable |
| 1. Vitamin B12 | Yes | | | No | | Not Applicable |
| 1. Vitamin D | Yes | | | No | | Not Applicable |
| 1. Do you feel that you know the fish consumption recommendations as they apply to you? | | | | | | |
| Yes | | No | | | Not Applicable | |
| The following questions relate to substances. | | | | | | |
| 1. Do you feel that you know the low-risk weekly alcohol intake guidelines as they apply to you? This includes amount of alcohol, frequency of drinking, and combinations with prescription and non-prescription drugs. | | | | | | |
| Yes | | No | | | Not Applicable | |
| 1. Do you feel that you know the low-risk daily alcohol intake guidelines as they apply to you? This includes amount of alcohol, frequency of drinking, and combinations with prescription and non-prescription drugs. | | | | | | |
| Yes | | No | | | Not Applicable | |
| 1. Do you feel that you know the recommended daily caffeine intake limits as they apply to you? | | | | | | |
| Yes | | No | | | Not Applicable | |
| 1. Do you feel that you know the recommended daily added sugar intake limits as they apply to you? | | | | | | |
| Yes | | No | | | Not Applicable | |
| 1. Do you feel that you know the recommendations about tobacco use as they apply to you? | | | | | | |
| Yes | | No | | | Not Applicable | |
| The following questions relate to lifestyle activities. | | | | | | |
| 1. Do you feel that you know the daily sleep recommendations as they apply to you? This includes quantity of sleep, quality of sleep, sleep hygiene, etc. | | | | | | |
| Yes | | No | | | Not Applicable | |
| 1. Do you feel that you know the weekly physical activity recommendations as they apply to you? This includes frequency, duration, intensity level, and types of activity. | | | | | | |
| Yes | | No | | | Not Applicable | |
| 1. Do you feel that you know the daily sedentary behaviour recommendations as they apply to you? | | | | | | |
| Yes | | No | | | Not Applicable | |
| 1. Do you feel that you know the daily recreational screen time recommendations as they apply to you? | | | | | | |
| Yes | | No | | | Not Applicable | |
| 1. Please reflect on your responses to Part 1. If you answered “Yes” to one or more of the questions in Part 1, what do you believe is the main reason why?   Example responses include:   - It’s a recommendation that has been important to my health or the health of someone close to me - I learned it during medical school studies - I learned it in undergraduate studies or earlier school - I learned it from my primary health care provider - I learned it from a public health campaign/poster/advertisement - I can’t remember how I know it - Any other reason you can think of | | | | | | |
| [free response] | | | | | | |
| 1. Please reflect on your responses to Part 1. If you answered “No” to one or more of the questions in Part 1, what do you believe is the main reason why?   Example responses include:   - I’ve never learned it - I learned it previously but have forgotten the specifics - Any other reason you can think of | | | | | | |
| [free response] | | | | | | |

| **Part 2:** The following questions relate to your achievement of recommendations for your health behaviours in an average week. You can respond as follows:   - Never (0 days in an average week) - Rarely (1-2 days in an average week) - Sometimes (3-4 days in an average week) - Usually (5-6 days in an average week) - Always (7 days in an average week) - If you know the recommendation(s) for the health behaviour but you don’t know how often you meet the recommendation(s), please respond with:   "I know the recommendations, but I don’t know how often I meet them”   - If you don’t know the recommendation(s) for the health behaviour, please respond with:   “I don’t know the recommendations” | | | | | | | |
| --- | --- | --- | --- | --- | --- | --- | --- |
| The following questions relate to diet. | | | | | | | |
| For questions 15-18, please estimate how many days you satisfy the recommendations for the following food-related health behaviours in an average week. | | | | | | | |
|  | Never  (0  days  per  week) | Rarely  (1-2 days  per week) | Some-times (3-4 days per week) | Usually  (5-6 days  per week) | Always  (7 days  per week) | I know the recomm-endations but I don’t know how often I meet them | I don’t know the recomm-endations |
| 1. Water intake |  |  |  |  |  |  |  |
| 1. Canada Food Guide | | | | | | | |
| 1. Fruits and vegetables |  |  |  |  |  |  |  |
| 1. Protein foods |  |  |  |  |  |  |  |
| 1. Whole grain foods |  |  |  |  |  |  |  |
| 1. Nutrients | | | | | | | |
| 1. Calcium |  |  |  |  |  |  |  |
| 1. Fats |  |  |  |  |  |  |  |
| 1. Fiber |  |  |  |  |  |  |  |
| 1. Folic Acid |  |  |  |  |  |  |  |
| 1. Iron |  |  |  |  |  |  |  |
| 1. Potassium |  |  |  |  |  |  |  |
| 1. Protein per kg of body weight |  |  |  |  |  |  |  |
| 1. Sodium |  |  |  |  |  |  |  |
| 1. Vitamin B12 |  |  |  |  |  |  |  |
| 1. Vitamin D |  |  |  |  |  |  |  |
| Other Foods | | | | | | | |
| 1. Fish |  |  |  |  |  |  |  |
| The following questions relate to substances. | | | | | | | |
| 1. Please estimate how many days you satisfy the recommendations for the following health behaviours in an average week. | | | | | | | |
|  | Never  (0  days  per  week) | Rarely  (1-2 days  per week) | Some-times  (3-4 days per week) | Usually  (5-6 days  per week) | Always  (7 days  per week) | I know the recomm-endations but I don’t know how often I meet them | I don’t know the recomm-endations |
| 1. Caffeine |  |  |  |  |  |  |  |
| 1. Added sugar |  |  |  |  |  |  |  |
| 1. Daily alcohol |  |  |  |  |  |  |  |
| 1. Tobacco use |  |  |  |  |  |  |  |
| 1. In an average week, do you satisfy the recommendations for weekly alcohol intake? | | | | | | | |
| Yes | | No | | I know the recommendations but I don’t know if I meet them in an average week | | I don’t know the recommendations | |
| The following questions relate to lifestyle activities. | | | | | | | |
| 1. Please estimate how many days you satisfy the recommendations for the following health behaviours in an average week. | | | | | | | |
|  | Never  (0  days  per  week) | Rarely  (1-2 days  per week) | Some-times  (3-4 days per week) | Usually  (5-6 days  per week) | Always  (7 days  per week) | I know the recomm-endations but I don’t know how often I meet them | I don’t know the recomm-endations |
| 1. Sleep |  |  |  |  |  |  |  |
| 1. Sedentary behaviour |  |  |  |  |  |  |  |
| 1. Recreational screen time |  |  |  |  |  |  |  |
| 1. In an average week, do you satisfy the weekly physical activity recommendations? | | | | | | | |
| Yes | | No | | I know the recommendations, but I don’t know if I meet them in an average week | | I don’t know the recommendations | |
| 1. Please reflect on your responses to Part 2. If you satisfy the recommendation(s) for one or more health behaviours in Part 2, what do you believe is the main reason why?   Example responses include:   - Doing it is rewarding to me - I need to do it in order to maintain basic healthy functioning - It is part of the lifestyle of my household/community - I’ve always done it - Any other reason you can think of | | | | | | | |
| [free response] | | | | | | | |
| 1. Please reflect on your responses to Part 2. If you don’t satisfy the recommendation(s) for one or more health behaviours in Part 2, what do you believe is the main reason why?   Example responses include:   - I don’t have enough time to prioritize it - I wouldn’t be able to function day-to-day if I tried to meet this recommendation (i.e., caffeine) - It is important to me, and I want to prioritize it, but I haven’t tried to yet - It is not important to me - I didn’t know it was a recommendation - It is not part of the lifestyle of my household/community - Any other reason you can think of | | | | | | | |
| [free response] | | | | | | | |

| **Part 3:** The following questions relate to the amount of time you spend engaging in certain behaviours in an average week. | | | |
| --- | --- | --- | --- |
| 1. Please estimate the number of hours you engage in each health behaviour in an average week. When estimating, please include the total time spent participating in the behaviour AND the time spent planning and preparing for it. | | | |
|  | Estimated Number of Hours | Unable to Estimate | Not Applicable |
| 1. Diet   (Meal planning, grocery shopping, meal preparation, eating, cleaning up, logging food in an app, etc) |  |  |  |
| 1. Physical activity   (Traveling to and from a venue if applicable, participating in the activity, showering afterward, logging activity in an app, etc) |  |  |  |
| 1. Sleep   (engaging in a sleep routine, lying in bed without screens before falling asleep, sleeping, lying in bed without screens after waking up, napping, etc ) |  |  |  |
| 1. Spiritual   (prayer, meditation, meeting with others in a spiritual community, etc) |  |  |  |
| 1. Medical and Mental Health   (taking medicines, recording blood pressure or blood sugars, therapies, counselling, health care practitioner appointments, etc) |  |  |  |
| 1. Hobbies   (extra-curricular activities including volunteering but excluding activities in parts a-f) |  |  |  |
| 1. Please reflect on your responses to Part 3. If you are happy with the amount of time you spend engaging in some or all of these behaviours, what do you believe is the main reason why you feel this way? | | | |
| [free response] | | | |
| 1. Please reflect on your responses to Part 3. If you are unhappy with the amount of time you spend engaging in some or all of these behaviours, what do you believe is the main reason why you feel this way? | | | |
| [free response] | | | |
